# The self-rated health status and key influencing factors in middle-aged and elderly: Evidence from the CHARLS

**DOI:** 10.1097/MD.0000000000027772

**Published:** 2021-11-19

**Authors:** Yu-Ling Zhang, Bin-Jiang Wu, Pei Chen, Ying Guo

**Affiliations:** a Department of Public Health, Jiangsu College of Nursing, Huai’an, Jiangsu Province, China; b Department of Basic Medicine, Jiangsu College of Nursing, Huai’an, Jiangsu Province, China; c Department of Medical Laboratory, Huai’an Maternal and Child Health Hospital, Jiangsu Province, China.

**Keywords:** China Health and Retirement Longitudinal Survey, chronic diseases, middle-aged and elderly, self-rated health

## Abstract

To evaluate the self-rate health (SRH) status and explore influence factors of middle-aged and elderly in China.

China Health and Retirement Longitudinal Survey was conducted in 2011, 2013, 2015 and 2018. Data of the China Health and Retirement Longitudinal Survey in 2018 was used in our study and a total of 17898 participants were included. SRH status was graded as “very good, good, average, bad, very bad.” Participants who answered “very good” and “good” were regarded as self-rated good health and who answered “average,” “bad” and “very bad” were regarded as self-rated poor health. Odds ratio and 95% confidence interval of Logistics regression were calculated to evaluate the correlation between SRH and chronic diseases, demographic characteristics and lifestyle of middle-aged and elderly participants.

A total of 4476 (25.01%) participants reported they had good health, and 13422 (74.99%) reported they had poor health. 9975 participants self-rated they had no chronic disease (55.73%), and 7923 (44.27%) participants self-rated they suffered from one and above chronic diseases. The prevalence of chronic diseases showed significant odds ratio and trend with SRH poor rate of participants. The more kinds of chronic diseases they suffered from, the poorer SRH was reported in middle-aged and elderly participants. Except for the chronic diseases, participants with higher age, living in rural, with high Center for Epidemiological Survey-Depression Scale score of depression and fewer time of physical activities also correlated with higher SRH (poor) rate.

The SRH (good) rate was very low in middle-aged and elderly, participants who accompanied with more kinds of chronic diseases, fewer physical activities, higher age and living in the rural had a worse health status. A more comprehensive and integrated health framework should be strengthened to improve the health of middle-aged and elderly in China.

## 1 Introduction

The aging population is developing rapidly in China. By the end of 2018, the number of elderly people over 60 years old has reached 249 million, accounting for 17.9% of the total population. It is estimated that the number of elderly people will exceed 400 million in 2050, and the aging level will reach more than 30%.^[1]^ With the aging of population, the prevalence of chronic diseases has increased rapidly, and the burden of diseases caused by age-related chronic non-communicable diseases will continue to increase.^[2]^ Chronic diseases are prevalent among older persons, 65% of U.S. men and 72% women with the age over 65 had two or more chronic illnesses in 2010.^[3]^

A lot of health indicators have been used to evaluate the health status of middle-aged and elderly, such as activity of daily living (ADL), quality of life, medical expense and so on. Self-rated health (SRH) is an individual's subjective evaluation and estimation of their own health status, which has been used as a screening tool to assess the health of older adults.^[4,5]^ Studies have shown that SRH status is closely related to the ADL and cognitive function of the elderly, which can predict adverse health outcomes and death risk.^[6,7]^ Poor SRH is significantly related to increased mortality.^[8]^ Ho SH ^[5]^ reported that chronic diseases, particularly stroke, were found to be a significant predictive factor related to poor health status. Due to the development of aging in China, the number of patients with chronic diseases is rising rapidly, especially in middle-aged and elderly. A lot of studies have discussed SRH issues primarily in terms of a single facet such as demographic factors, healthcare utilization or chronic diseases and that have seldom been investigated across these factors, particularly with regard to health status related to middle-aged and elderly. To fill this gap, our study aim to evaluate the SRH and find out the key factors which seriously influence the SRH status of middle-aged and elderly in China, and help to formulate more effective programs to improve the awareness and management of chronic diseases in China and other developing countries based on the data of the China Health and Retirement Longitudinal Survey (CHARLS) in 2018.

The data used in this research was obtained from CHARLS. First, we sorted out the data of the CHARLS in 2018 survey to remove invalid questionnaires and acquired valid results of SRH status, chronic diseases, physical activities, gender and other data in middle-aged and elderly participants. Second, we analyzed the relationship between SRH status and chronic diseases, physical activities, depression in middle-aged and elderly. Third, a multiple regression analysis was used to analyze the key influencing factors of poor SRH in middle-aged and elderly.

## 2 Methods

### 2.1 Participants and data

CHARLS is a large-scale interdisciplinary survey project sponsored by the National Development Research Institute of Peking University and jointly implemented by the China Social Science Survey Center of Peking University and the Youth League Committee of Peking University. It is a major project funded by the National Natural Science Foundation of China. It aims to collect a set of high-quality micro data representing the families and individuals of the middle-aged and elderly people aged 45 and above in China for the analysis of China's aging population and promoting interdisciplinary research on aging. CHARLS conducted surveys and interviews in 150 counties and 450 communities (villages) of 28 provinces (autonomous regions and municipalities) in 2011, 2013, 2015 and 2018 respectively. By the time of completion of the nationwide follow-up in 2018, its samples had covered 19000 respondents in a total of 12400 families. Multi-stage Probability Proportionate to Size Sampling method was adopted for sampling through the four stages of county (district)-village (resident)-household-individual. In our study, the data of CHARLS in 2018 was used. All data collected in the CHARLS are maintained at the National School of Development of Peking University. All the data can be found at http://charls.pku.edu.cn. The CHARLS was approved by the Ethical Review Committee of Peking University, and all participants signed an informed consent form before the start of the investigation.

### 2.2 Variables

#### 2.2.1 Self-rated health status

Results of SRH were acquired through a single choice question “what do you think of your health status.” The options were “very good, good, average, bad, very bad.” In the analysis, the five categories of SRH were transformed into two categories of variables, namely, self-rated good health (answering “very good” and “good”) and self-rated poor health (answering “average,” “bad” and “very bad”).^[9]^

#### 2.2.2 Chronic diseases

A total of 14 chronic diseases were included in the questionnaire, they were hypertension, dyslipidemia, diabetes, cancer, chronic lung diseases, liver disease, heart disease, stroke, kidney disease, stomach disease, emotional disease, memory-related disease, arthritis and asthma. In the questionnaire, participants were asked if a doctor had ever diagnosed them that they had suffered from hypertension, diabetes and other 12 chronic diseases. Participants who answered “yes” were identified as self-rated chronic diseases, and the others were no self-rated chronic diseases.

#### 2.2.3 Physical activities

Physical activities were divided into three types, vigorous, moderate and light. Vigorous physical activities made participants breathe much harder than normal, including heavy lifting, aerobics, fast bicycling and so on. Moderate physical activities made participants somewhat harder than normal, including carrying light loads, bicycling at a regular pace, practicing Taijiquan and so on. Light physical activities include walking from place to place and other walking solely for recreation, exercise or leisure. The time spent on physical activities was divided into less than 30 minutes (<30 min), between 30 minutes and 2 hours (30 min – 2 h), more than 2 hours (≥ 2 h).

#### 2.2.4 Depression

Depression was examined by the Center for Epidemiological Survey, Depression Scale (CES-D), which had 10 items. 0 = hardly like this (less than 1 day), 1 = a little like this (1-2 days), 2 = a lot like this (3-4 days), 3 = almost like this (5-7 days). The total score ranged from 0 to 30, participants with total score <10 were defined as “without depressive symptoms,” with 10-20 score as “with depressive symptoms;” 20 or more as “with severe depressive symptoms.”

### 2.3 Statistical analysis

STATA 16.0 software was used to conduct statistical analysis. The mean and standard deviation were used to describe quantitative variables, and rate or composition ratio were used to describe categorical variables. *t* test was used to analyze the mean difference of quantitative variables and χ2 test was used to analyze rate or composition ratio difference of categorical variables. Multiple logistic regression was used to calculate the odds ratio and 95% confidence interval for SRH influence factors. α=0.05 was used as the test level, when *P* value was less than .05 (two-sided test), the difference was considered to be statistically significant.

## 3 Results

### 3.1 Demographic characteristic

**Figure 1:**
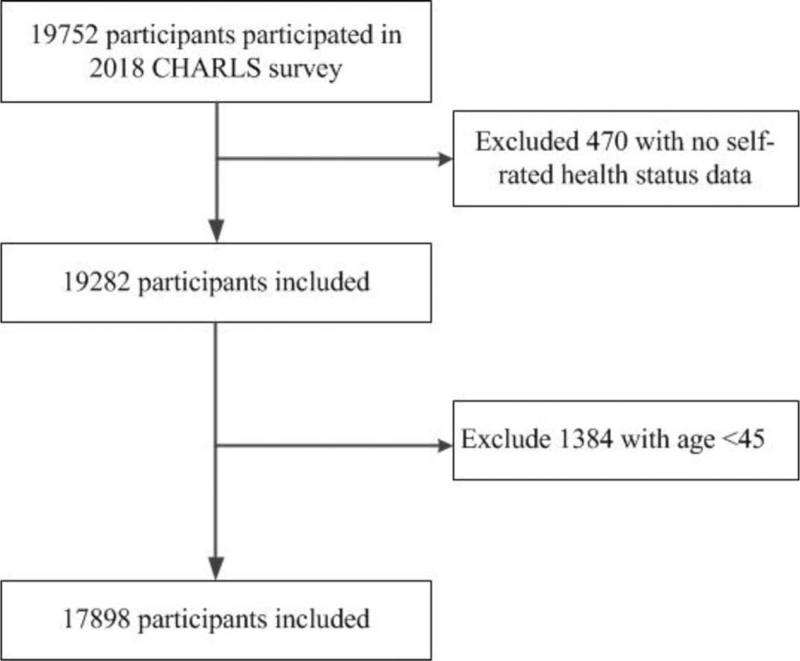
Flow chat of participants screening.

A total of 17898 participants were included in our study, including self-rated chronic diseases participants 7923 and no self-rated disease participants 9975. Significant differences were observed between self-rated chronic diseases group and no self-rated chronic diseases group in age, location of residence and marital status, *P* < .05. (Fig. 1 and Table 1).

**Table 1:** Characteristics of middle-aged and elderly participants of the CHARLS in 2018.

		SRH (good)		SRH (poor)		
Characteristic	Number of participants	n	%	n	%	*P* value
Age (yr)						
≥45–<55	4430	1416	31.96%	3014	68.04%	
≥55–<65	6025	1587	26.34%	4438	73.66%	<.01
≥65–<75	5218	1045	20.03%	4173	79.97%	
≥75	2225	428	19.24%	1798	80.81%	
Gender						
Male	8497	2351	27.67%	6146	72.33%	<.01
Female	9401	2125	22.60%	7276	77.40%	
Location of residence						
City	4453	1329	29.85%	3124	70.15%	<.01
Rural	13445	3147	23.41%	10298	76.59%	
Marital status						
Married	15417	3959	25.68%	11458	74.32%	<.01
Never married or divorced	2481	517	20.84%	1964	79.16%	
Education						
Primary and below	15606	3658	23.44%	11948	76.56%	<.01
Secondary and above	2292	818	35.69%	1474	64.31%	

CHARLS = China Health and Retirement Longitudinal Survey, SRH = self-rated health.

### 3.2 SRH status in middle-aged and elderly

A total of 13422 announced they had poor health in 17898 participants, the rate was 74.99%. 3234 participants in self-rated no chronic diseases group (9975) reported they had good health (32.42%), 924 participants in self-rated one chronic disease (4930) reported they had good health (18.74%), 240 participants in self-rated two chronic diseases (1850) reported they had good health (12.97%), 78 participants in self-rated three chronic diseases reported they had good health (6.82%). Compared with no chronic disease, significant difference was observed in one, two and three and above chronic diseases group, *P* < .05.(Table 2).

**Table 2:** SRH status in middle-aged and elderly.

Group	Number of participants	SRH good	Rate	*P* value
No chronic disease	9975	3234	32.42%	<.01
One chronic disease	4930	924	18.74%	
Two chronic disease	1850	240	12.97%	
Three and above chronic disease	1143	78	6.82%	
Total	17898	4476	25.01%	

SRH = self-rated health.

**Figure 2:**
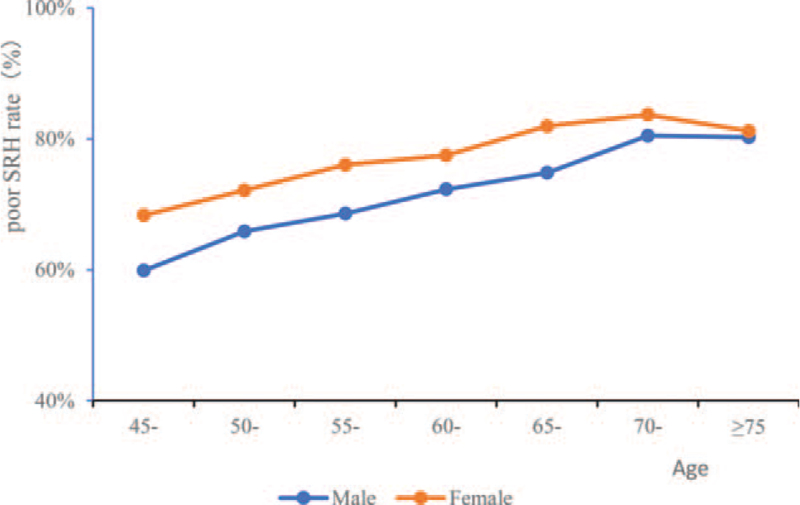
Occurrence probability of poor SRH in middle-aged and elderly by gender. SRH = self-rated health.

**Figure 3:**
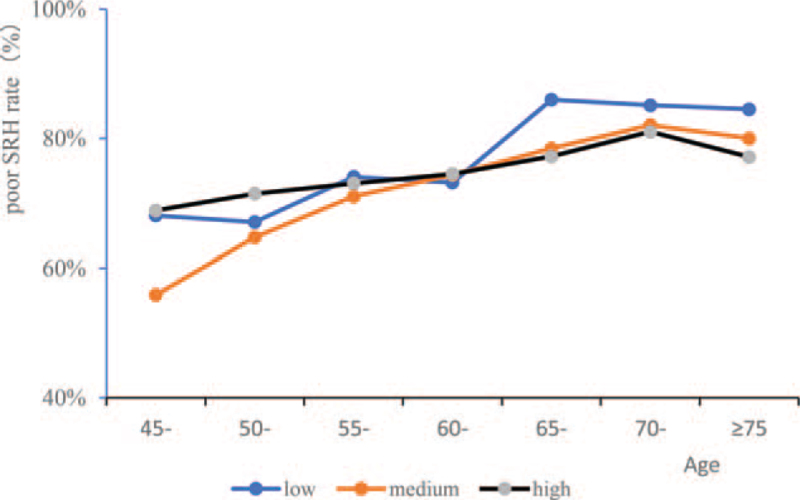
Occurrence probability of poor SRH in middle-aged and elderly by physical activities. SRH = self-rated health.

Line funnel were drawn to predict probability of self-rated poor health in middle-aged and elderly for age incremented by 5 years stratified by gender and physical activities. Both male and female had increasing poor SRH rate with age, and female has a higher poor SRH rate than male (Fig. 2). Below the age of 60, low, medium and high physical activities had no obvious different effects on poor SRH rate. Above the age of 60, participants with more physical activities have a lower poor SRH rate (Fig. 3).

### 3.3 Relationship between SRH status and the Charlson comorbidity scores of chronic diseases in middle-aged and elderly

To evaluate the influence of chronic diseases on SRH, we evaluated the Charlson comorbidity scores of chronic diseases. In the SRH (good) group, 3379 (75.49%) participants with the Charlson comorbidity scores “0,” 1078 (24.08%) participants with the Charlson comorbidity scores “1–3,” 19 (0.42%) participants with the Charlson comorbidity scores “≥4.” In the SRH (poor) group, 7264 (54.12%) participants with the Charlson comorbidity scores “0,” 5893 (43.91%) participants with the Charlson comorbidity scores “1–3,” 265 (1.97%) participants with the Charlson comorbidity scores “≥4.” The rate of participants with higher Charlson comorbidity scores was higher in SRH (poor) than that in SRH (good) group, *P* < .05. (Table 3).

**Table 3:** The Charlson comorbidity scores of different SRH status in middle-aged and elderly.

Group	Scores	Number of participants	Rate	*P* value
SRH (good)	0	3379	75.49%	<.01
	1–3	1078	24.08%	
	≥4	19	0.42%	
SRH (poor)	0	7264	54.12%	
	1–3	5893	43.91%	
	≥4	265	1.97%	

SRH = self-rated health.

### 3.4 Depression and physical activities in middle-aged and elderly

The CES-D depression scores were significantly lower in SRH (good) group (7.84 ± 0.07) than that in SRH (poor) group (10.09 ± 0.05). The participants with SRH (good) spent more time on daily physical activities than participants with SRH (poor). (Table 4).

**Table 4:** Relationship between SRH and depression, physical activity.

	Depression		Physical activity			
Group	n	scores	n	<30min/d	30min-2h/d	≥2h/d
SRH (good)	4468	7.84 ± 0.07	4161	227 (5.46%)	1465 (35.21%)	2469 (59.34%)
SRH (poor)	13400	10.09 ± 0.05	12156	816 (6.71%)	4083 (33.59%)	7257 (59.70%)
Total	17868^∗^	9.52 ± 0.04	16317^†^	1043	5548	9726

SRH = self-rated health.

∗ 30 participants did not provide the result of decompression.

† 1581 participants did not provide the result of physical activity.

The CES-D depression scores in no chronic disease were 8.92 ± 5.29 which was statistically lower than that in one chronic disease group (9.86 ± 5.58), two chronic diseases group (10.68 ± 5.77) and three and above chronic diseases group (11.46 ± 6.06), significantly statistical difference was observed between each group, *P* < .05. Participants with no chronic diseases group spent more time on daily physical activities than participants with chronic diseases, significantly statistical difference was also observed between each group, *P* < .05. (Table 5).

**Table 5:** Relationship between chronic diseases and depression, physical activity.

	Depression		Physical activity^†^		
Group	n	scores	<30min/d	30min-2h/d	≥2h/d
No chronic disease	9960	8.92 ± 5.29	551 (6.03%)	2970 (32.49%)	5620 (61.48%)
One chronic disease	4923	9.86 ± 5.58	293 (6.49%)	1586 (35.12%)	2637 (58.39%)
Two chronic disease	1847	10.68 ± 5.77	120 (7.27%)	584 (35.39%)	946 (57.33%)
Three and above	1138	11.46 ± 6.06	79 (7.82%)	408 (40.40%)	523 (51.78%)
Total	17868^∗^	9.52 ± 5.53	1043	5548	9726

∗ 30 participants did not provide the result of decompression.

† 1581 participants did not provide the result of physical activity.

### 3.5 Influence factors of poor SRH in middle-aged and elderly

Logistics regression was used to analyze the factors which influenced SRH in middle-aged and elderly. In the results of multiple logistics regression, age, gender, location of residence, education, depression and chronic diseases were key factors which significantly influenced the SRH of middle-aged and elderly. The chronic diseases, age and living in the rural were important influencing factors which positively affected self-rated poor health. The participants had a higher self-rated poor health rate if they had higher age, one or more chronic diseases and living in the rural. On the contrary, higher education and physical activities negatively affected self-rated poor health. The participants had a lower self-rated poor health rate if they had higher education or more than 30 minutes physical activities every day. The female participants also had a higher self-rated poor health rate than male participants (Tables 6 and 7).

**Table 6:** Influence factors of self-rated poor health in middle aged and elderly.

Variable	OR	95%CI	*P* value
Age			
≥45-<55			
≥55-<65	1.15	1.10–1.20	<.01
≥65-<75	1.23	1.20–1.27	<.01
≥75	1.19	1.15–1.22	<.01
Gender			
Male			
Female	1.31	1.22–1.40	<.01
Location of residence			
City			
Rural	1.40	1.29–1.52	<.01
Marital status			
Married			
Never married or divorced	1.04	0.92–1.16	.56
Education			
Primary and below			
Secondary and above	0.55	0.50–0.61	<.01
Depression	1.09	1.08–1.10	<.01
Physical activity			
<30min/d			
30min-2h/d	0.77	0.66–0.91	<.01
≥2h/d	0.90	0.84–0.98	.01
Chronic disease			
No chronic disease			
One chronic disease	2.08	1.91–2.26	<.01
Two chronic diseases	1.79	1.67–1.93	<.01
Three and above	1.87	1.73–2.02	<.01

95%CI = 95% confidence interval, OR = odds ratio.

**Table 7:** Multiple logistics regression model about the influence factors of self-rated poor health in middle aged and elderly.

Variables	Regression coefficient β	95%CI	*P* value
Age	0.39	0.31–0.47	<.01
Gender	0.15	0.07–0.22	<.01
Location of residence	0.31	0.22–0.40	<.01
Marital status	−0.03	−0.14–0.09	.68
Education	−0.38	−0.49--0.27	<.01
Depression	0.07	0.07–0.08	<.01
Physical activity	−0.05	−0.12–0.01	.09
Chronic disease	0.61	0.56–0.67	<.01

95%CI = 95%confidence interval.

## 4 Discussion

Various health indicators have been used to examine health status, including morbidity, ADL, quality of life. However, it is difficult to assess overall health of participants using these multiple indicators.^[10]^ SRH has been regarded as an increasingly common measure for public health monitoring which is a strong independent predictor of mortality after accounting for objective health status, lifestyle risk factors and socio-demographic characteristics, and it also provides a suitable and inexpensive method of assessing an individual's health.^[11]^ Various socio-demographic, health and lifestyle determinants of SRH have been identified in different populations, such as aging, sleeping, physical activities and so on.

In recent years, with the aging of population development, chronic diseases have become the main factor affecting the health of the population in China. More than half (51.8%) of adults had at least 1 of 10 selected diagnosed chronic conditions (arthritis, cancer, chronic obstructive pulmonary disease, coronary heart disease, current asthma, diabetes, hepatitis, hypertension, stroke, and weak or failing kidneys), and 27.2% of US adults had multiple chronic conditions.^[12]^ Among the chronic diseases, the prevalence of hypertension, diabetes and other diseases increased significantly. In our study, we found that 44.27% participants reported that they suffered from one or more chronic diseases. The rate of self-reported one chronic disease was 27.54%, two chronic diseases 10.34%, three and above chronic diseases 6.39% respectively. These results were consistent with the analysis report of the Fifth National Health Service Survey in 2013, the prevalence of chronic diseases in China was 29.50% in aged 45 to 54, 52.60% in aged 55 to 64 and 78.40% in aged over 65.^[13]^ Most middle-aged and elderly self-rated themselves unhealthy, the self-rate health rate was only 25.01%. The total scores of the Charlson comorbidity in SRH good group was significantly lower than that in SRH poor group, chronic diseases and their complications were key influencing factors affecting good SRH in middle-aged and elderly. According to the logistics regression results, chronic disease was also one of the most important factors which caused self-rated poor health in middle-aged and elderly. Due to long course and poor prognosis, chronic diseases not only lead to physical and functional damage, but also have long-term and serious negative effects on patients’ psychological status and social adaptability. With the changing of life style and trend of aging, the prevalence of chronic diseases significantly increases, the loss of quality of life and the burden of disease caused to the society are becoming more and more serious.

Participants with higher age who are usually accompanied by chronic diseases and functional decline often report poorer SRH and encompassing quality of life.^[14]^ In our study, we found that higher age was correlated with both chronic diseases and poor SRH. Both male and female had increased poor SRH rate with age increasing, and the female participants had a higher poor SRH rate than male before the age of 75. After the age of 75, the difference of poor SRH rate between female and male participants gradually disappeared. Some studies reported that older adults, especially those who were widowed, experience worse health than those whose spouses were still alive. Widowed older adults experienced the effects of bereavement, which had a negative impact on their health and survival rate.^[15–17]^ In our study, we found that the prevalence of chronic diseases was lower in married than that in never married or divorced, but different SRH rate was not observed between the two groups. Higher odds of poor SRH were found among people from low socio-economic status.^[18]^ We also found that participants who were living in rural and with lower education announced a higher rate of poor SRH. Strategies and programs that aim to improve health conditions in rural populations should be paid for more attention. Depression was often regarding as one of the influencing factors which decrease the SRH status.^[19]^ In our study, we found that depression correlated with both poor SRH and chronic diseases. The CES-D depression scores was significantly lower in good SRH group than that in poor SRH group, and the participants with chronic diseases had higher depression scores than that in no chronic disease participants, and participants with three and above had the highest depression scores. The logistics regression results indicated depression was one of the influencing factors which affected SRH status in middle-aged and elderly. Physical activities were often correlated with the activities of daily living in elderly, previous studies have reported it related to better self-rated wellbeing, and more frequent physical activities were linearly associated with better self-rated wellbeing.^[20,21]^ In our study, physical activities were divided into three levels, low (<30 min/d), medium (30min-2 h/d) and high (≥2 h/d). Results indicated that the time spent on physical activities were positively correlated with the rate of self-rated good health. Below the age of 60, low, medium and high physical activities had no obvious different effects on poor SRH rate. Above the age of 60, participants with more physical activities have a lower poor SRH rate.

## 5 Conclusion and limitations

Our study provided a large sample survey of SRH status in middle-aged and elderly and provided evidence of a significant association between SRH status and chronic diseases, physical activities, depression, location of residence, marital status, and so on. We found that the self-rated good health rate was very low in middle-aged and elderly and participants who accompanied with more kinds of chronic disease, fewer physical activities, living in the rural and higher-age had a worse health status. These findings identified that chronic diseases, life situation and psychological status correlated strongly with self-rated health for middle-aged and elderly. The middle-aged and elderly, especially these with chronic diseases, should be paid more attention not only physically but also psychologically to improve their health status and life quality. A more comprehensive and integrated health framework should be strengthened to improve the health status of middle-aged and elderly. Self-rated health may be used as a screening tool to evaluate the health status of middle-aged and elderly and the results may be taken as a reference by public health officials to promote healthy aging.

Our study was also affected by a number of limitations. First, this study was a cross-sectional design, which could not determine the causal relationship between the SRH status and the main influencing factors, the results needed to be verified by another prospective studies. Second, our demonstrated relationship between SRH status and chronic diseases, physical and psychological conditions of middle-aged and elderly were limited in the country of China. A comprehensive and multidimensional self-rated evaluation model should be established to verify our results, because of some bias might exist among different countries. Third, different chronic diseases have many similar symptoms, such as chronic pain. The relationship between SRH status and chronic diseases in middle-aged and elderly could be further analyzed through the symptoms of chronic diseases. Fourth, because the CHARLS was a cross sectional investigation and the chronic diseases often have a long disease course, recall bias is inevitable. Some participants tend to have a profound impact on recent events and might over-feedback during the questionnaire survey, therefore, some selection bias might also exist in our research.

## Acknowledgments

The authors thank all the participants in CHARLS team for their time and effort devoted to the project.

## Author contributions

**Data curation:** Pei Chen, Ying Guo.

**Formal analysis:** Ying Guo.

**Methodology:** Bin-Jiang Wu.

**Project administration:** Yu-Ling Zhang.

**Resources:** Bin-Jiang Wu.

**Writing – original draft:** Yu-Ling Zhang.

**Writing – review & editing:** Yu-Ling Zhang, Pei Chen.

## References

[1] Du P Zhai ZW Chen W. Centennial trend of population aging in China. Population Res 2005;29:90–3. Chinese.

[2] Prince MJ Wu F Guo Y. The burden of disease in older people and implications for health policy and practice. Lancet 2015;385:549–62. 25468153 10.1016/S0140-6736(14)61347-7

[3] Ward BW Schiller JS. Prevalence of multiple chronic conditions among US adults: estimates from the National Health Interview Survey, 2010. Prev Chronic Dis 2013;10:E65. 23618545 10.5888/pcd10.120203 PMC3652717

[4] Moriconi PA Nadeau L. A cross-sectional study of self-rated health among older adults: association with drinking profiles and other determinants of health. Curr Gerontol Geriatr Res 2015;2015:352947. 26843861 10.1155/2015/352947 PMC4710937

[5] Ho SH. Correlations among self-rated health, chronic disease, and healthcare utilization in widowed older adults in Taiwan. J Nurs Res 2018;26:308–15. 29219938 10.1097/jnr.0000000000000248

[6] Mossey JM Shapiro E. Self-rated health: a predictor of mortality among the elderly. Am J Public Health 1982;72:800–8. 7091475 10.2105/ajph.72.8.800 PMC1650365

[7] Ghorbani Saeedian R Nagyova I Klein D. Self-rated health mediates the association between functional status and health-related quality of life in Parkinson's disease. J Clin Nurs 2014;23:1970–7. 24354845 10.1111/jocn.12442

[8] Benjamins MR Hummer RA Eberstein IW Nam CB. Self-reported health and adult mortality risk: an analysis of cause-specific mortality. Soc Sci Med 2004;59:1297–306. 15210100 10.1016/j.socscimed.2003.01.001

[9] Cislaghi B Cislaghi C. Self-rated health as a valid indicator for health equity analyses: evidence from the Italian health interview survey. BMC Public Health 2019;19:533. 31072306 10.1186/s12889-019-6839-5 PMC6509759

[10] Yamada C Moriyama K Takahashi E. Self-rated health as a comprehensive indicator of lifestyle-related health status. Environ Health Prev Med 2012;17:457–62. 22426707 10.1007/s12199-012-0274-x PMC3493633

[11] Frange C de Queiroz SS da Silva Prado JM Tufik S de Mello MT. The impact of sleep duration on self-rated health. Sleep Sci 2014;7:107–13. 26483912 10.1016/j.slsci.2014.09.006 PMC4521644

[12] Boersma P Black LI Ward BW. Prevalence of multiple chronic conditions among US Adults, 2018. Prev Chronic Dis 2020;17:E106. 32945769 10.5888/pcd17.200130 PMC7553211

[13] Statistical information center of health and Family Planning Commission of China. Analysis report of the Fifth National Health Service Survey in 2013 [R]. 2015.20-30.

[14] Janssen I Ross R. Linking age-related changes in skeletal muscle mass and composition with metabolism and disease. J Nutr Health Aging 2005;9:408–19. 16395513

[15] Perrig-Chiello P Spahni S Höpflinger F Carr D. Cohort and gender differences in psychosocial adjustment to later-life widowhood. J Gerontol B Psychol Sci Soc Sci 2016;71:765–74. 25731182 10.1093/geronb/gbv004

[16] Vable AM Subramanian SV Rist PM Glymour MM. Does the “widowhood effect” precede spousal bereavement? Results from a nationally representative sample of older adults. Am J Geriatr Psychiatry 2015;23:283–92. 24974142 10.1016/j.jagp.2014.05.004 PMC5511695

[17] Spahni S Bennett KM Perrig-Chiello P. Psychological adaptation to spousal bereavement in old age: the role of trait resilience, marital history, and context of death. Death Stud 2016;40:182–90. 26745606 10.1080/07481187.2015.1109566

[18] Caicedo-Velásquez B Restrepo-Méndez MC. The role of individual, household, and area of residence factors on self-rated health in Colombian adults: a multilevel study. Biomedica 2020;40:296–308. 32673458 10.7705/biomedica.4818 PMC7505506

[19] Yao Y Liu M Yang SS. Study on self-rated health and related factors in centenarians in Hainan province. Zhonghua Liu Xing Bing Xue Za Zhi 2018;39:264–7. Chinese. 29609236 10.3760/cma.j.issn.0254-6450.2018.03.003

[20] Peralta M Martins J Gómez Chávez F Cortés Almanzar P Marques A. Self-rated wellbeing and physical activities associations in European older adults. Eur J Sport Sci 2018;18:1038–44. 29738679 10.1080/17461391.2018.1469672

[21] Marques A Peralta M Martins J Catunda R Matos MG Saboga Nunes L. Associations between physical activities and self-rated wellbeing in European adults: a population-based, cross-sectional study. Prev Med 2016;91:18–23. 27471019 10.1016/j.ypmed.2016.07.021

